# Sperm Quality during Storage Is Not Affected by the Presence of Antibiotics in EquiPlus Semen Extender but Is Improved by Single Layer Centrifugation

**DOI:** 10.3390/antibiotics7010001

**Published:** 2017-12-21

**Authors:** Ziyad Al-Kass, Joachim Spergser, Christine Aurich, Juliane Kuhl, Kathrin Schmidt, Anders Johannisson, Jane M. Morrell

**Affiliations:** 1 Clinical Sciences, Swedish University of Agricultural Sciences (SLU), SE-75007 Uppsala, Sweden; ziyad.al.kass@slu.se or ziyadalkass@gmail.com; (Z.A.-K.); Anders.Johannisson@slu.se (A.J.); 2 Department of Surgery and Theriogenology, College of Veterinary Medicine, University of Mosul, Mosul 41002, Iraq; 3 Institute of Microbiology, Vetmeduni Vienna, 1210 Vienna, Austria; joachim.spergser@vetmeduni.ac.at; 4 Centre for Artificial Insemination and Embryo Transfer, Vetmeduni Vienna, 1210 Vienna, Austria; Christine.Aurich@vetmeduni.ac.at (C.A.); juliane.kuhl@vetmeduni.ac.at (J.K.); kathrin.schmidt@vetmeduni.ac.at (K.S.)

**Keywords:** pony stallions, bacteria, semen evaluation

## Abstract

Contamination of semen with bacteria arises during semen collection and handling. This bacterial contamination is typically controlled by adding antibiotics to semen extenders but intensive usage of antibiotics can lead to the development of bacterial resistance and may be detrimental to sperm quality. The objective of this study was to determine the effects of antibiotics in a semen extender on sperm quality and to investigate the effects of removal of bacteria by modified Single Layer Centrifugation (MSLC) through a colloid. Semen was collected from six adult pony stallions (three ejaculates per male). Aliquots of extended semen were used for MSLC with Equicoll, resulting in four treatment groups: control and MSLC in extender with antibiotics (CA and SA, respectively); control and MSLC in extender without antibiotics (CW and SW, respectively). Sperm motility, membrane integrity, mitochondrial membrane potential and chromatin integrity were evaluated daily by computer-assisted sperm analysis (CASA) and flow cytometry. There were no differences in sperm quality between CA and CW, or between SA and SW, although progressive motility was negatively correlated to total bacterial counts at 0 h. However, MSLC groups showed higher mean total motility (P < 0.001), progressive motility (P < 0.05), membrane integrity (P < 0.0001) and mitochondrial membrane potential (P < 0.05), as well as better chromatin integrity (P < 0.05), than controls. Sperm quality remained higher in the MSLC groups than controls throughout storage. These results indicate that sperm quality was not adversely affected by the presence of antibiotics but was improved considerably by MSLC.

## 1. Introduction

Semen often contains bacteria due to contamination during collection and processing. Bacteria originate from the penis and prepuce of the stallion, the environment, and from semen handling. These bacteria may cause endometritis in inseminated mares [1], contribute to decreased semen quality [2], and affect fertility [3]. To avoid such problems, antibiotics are added to semen extenders. However, excessive use of antibiotics may lead to the development of antibiotic resistance [4]. In addition, antibiotics in the semen extender may be detrimental to semen characteristics during cooled-storage [5,6]. Therefore, controlling bacteria with antibiotics may not be desirable.

No single method of sperm quality evaluation can be used on its own to predict fertility [7]. Previously, sperm number, sperm motility and morphology were used to evaluate “sperm quality”, but now additional assays are available to evaluate sperm functionality. Computer Assisted Sperm Analyzers for motility (CASA) and Flow cytometry (FC) enable additional parameters of sperm quality to be tested [8]. The CASA is able to analyze sperm motility and kinematics [9]. Flow cytometry can be used to evaluate sperm plasma membrane integrity, acrosome integrity, chromatin integrity, mitochondrial status and other parameters [10]. These assays provide an objective means of analyzing several thousand spermatozoa per sample, enabling an objective assessment of the effects of the presence of bacteria, or of antibiotics, on sperm quality to be made.

An alternative method to control bacteria in semen would be to remove them physically. The technique of Single Layer Centrifugation (SLC) through a colloid was shown to improve sperm quality [11] and to separate spermatozoa from seminal plasma. It has also been reported to separate spermatozoa from bacteria in boar semen [12]. A modification of this technique, MSLC, was shown to reduce bacterial contamination in stallion semen [11,13] but did not remove all the bacteria. Therefore, it is important to investigate the effects of these residual bacteria on sperm quality after SLC and to determine whether antibiotics are needed to control such contamination.

The aims of this study were (1) to determine the effect of antibiotics on spermatozoa, by comparing sperm quality in extender with and without antibiotics; and (2) to investigate the effect of removal of bacteria on sperm quality, by monitoring sperm quality in SLC-selected samples and controls during storage for several days.

## 2. Materials and Methods

### 2.1. Animals

Semen was collected from six adult pony stallions (5–25 years old). The animals were housed according to standard husbandry practices at the Center for Artificial Insemination and Embryo Transfer, Vetmeduni Vienna, Austria. The semen collection was approved by the competent authority for animal experimentation (Austrian Federal Ministry for Science and Research, license number BMWFW-68.205/0150-WF/V/3b/2015).

### 2.2. Semen

#### 2.2.1. Semen Collection

The ejaculates (3 per male) were collected using a sterilized Hannover artificial vagina after the stallion had mounted a phantom. Each ejaculate was split into two parts and extended in EquiPlus (Minitüb, Tiefenbach, Germany), either with (A) or without (W) antibiotics, to give a sperm concentration of 100 × 10^6^/mL. The extender is a commercially available product; according to the manufacturer, it contains lincomycin 0.015 g and spectinomycin 0.025 g per 100 mL with pH 6.8 ± 0.2 and 320 ± mOs·mol/L osmotic pressure.

#### 2.2.2. Semen Preparation with Modified Single Layer Centrifugation

**Figure 1:**
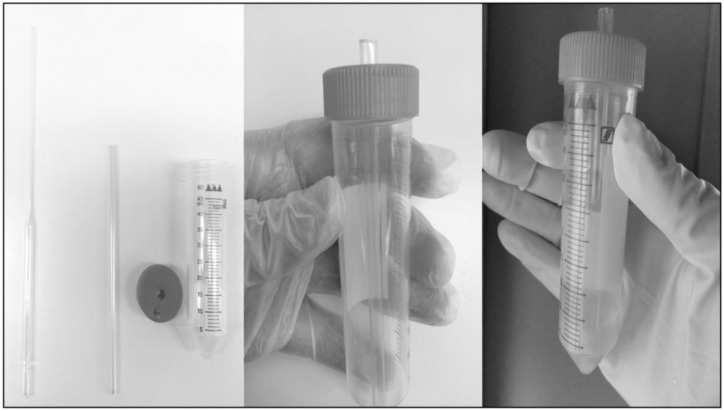
Modified Single Layer Centrifugation (MSLC). A 50 mL sterile tube with a sterile 5 mL plastic inner tube inserted through the middle of the cover, and a small hole that had previously been made at the edge of the lid. The colloid is poured into the 50 mL tube, the lid containing the insert is screwed on, and the semen samples are added through the small hole near the edge of the lid. After centrifugation, the sperm pellet can be retrieved easily by passing a long Pasteur pipette through the plastic insert.

Aliquots of extended semen were used for MSLC with Equicoll [11] under aseptic conditions. Briefly, the colloid (15 mL) was poured into a 50 mL sterile tube and a sterile 5 mL plastic tube (the sheath from a Cytology Brush; Minitube, Celadice, Slovakia) was inserted through the middle of the cover [13]. An aliquot (15 mL) of semen, adjusted to a sperm concentration of 100 × 10^6^/mL, was pipetted on top of the colloid through a small hole that had previously been made at the edge of the lid. The tube was centrifuged at 300× *g* for 20 min using a swing-out rotor. The sperm pellet was then recovered using a long Pasteur pipette passed through the tube insert and was resuspended in the appropriate extender (Figure 1). Four treatment groups were formed: control and MSLC in EquiPlus with antibiotics (CA and SA, respectively); control and MSLC in EquiPlus without antibiotics (CW and SW, respectively). The sperm concentration was adjusted to 50 × 10^6^/mL in all samples, which were then stored at 6 °C for 96 h.

### 2.3. Evaluation

#### 2.3.1. Sperm Concentration

Sperm concentration was measured using a Nucleocounter-SP 100 (Chemometec, Allerød, Denmark) as follows: 50 µL semen sample were mixed with 5 mL reagent S100 (Chemometic, Allerød, Denmark) and this mixture was loaded into a cassette containing the fluorescent dye propidium iodide (PI). The cassette was inserted into the fluorescence meter, which displayed the sperm concentration after 30 s.

#### 2.3.2. Computer-Assisted Sperm Analysis (CASA)

Motility evaluation was performed using a SpermVision analyzer (Minitüb GmbH, Tiefenbach, Germany), connected to an Olympus BX 51 microscope (Olympus, Tokyo, Japan) with a heated stage (38 °C); samples were equilibrated to room temperature before motility analysis. Sperm motility was analyzed in eight fields (at least 1000 spermatozoa in total) using the SpermVision software program with settings adjusted for stallion spermatozoa. Spermatozoa were considered as immotile if VAP < 20; locally motile if VAP > 20 and < 30, STR < 0.5, VCL < 9. The following kinematics were assessed at 0, 24, 48, 72 and 96 h: total motility (TM, %), progressive motility (PM, %), velocity of the average path (VAP, µm/s), curvilinear velocity (VCL, µm/s), straight line velocity (VSL, µm/s), straightness (STR, %), linearity (LIN, %), wobble (WOB, %) lateral head displacement (ALH, µm), beat cross frequency (BCF, Hz).

#### 2.3.3. Membrane Integrity

Aliquots of each sample were adjusted to a sperm concentration of approximately 2 × 10^6^ spermatozoa/mL with CellWASH (Becton Dickinson, San José, CA, USA). Assessment of plasma membrane integrity was made in 300 µL of the diluted sample after staining with 0.6 μL of 0.02 μM SYBR14 and 3 μL of 12 μM PI (Live-Dead Sperm Viability Kit L-7011; Invitrogen, Eugene, OR, USA) and incubating for 10 min at 37 °C [14]. The green and red fluorescence, as well as forward and side scatter, were measured using a FACSVerse™ flow cytometer (BD Biosciences). Membrane integrity was assessed at 24, 48, 72 and 96 h, using the classification intact membranes (SYBR14 positive, PI negative) and damaged membranes (SYBR14 positive or negative/PI positive). For the purposes of this study, only the proportion of spermatozoa with intact membranes was reported.

#### 2.3.4. Assessment of Mitochondrial Membrane Potential (MMP)

Aliquots of the diluted sperm samples (1000 µL) were stained with 0.5 µL of 3 mM JC-1, followed by incubation at 37 °C for 30 min and evaluation by FC [15]. The green and orange fluorescence, as well as forward and side scatter were measured using a FACSVerse™ flow cytometer. Proportions of the sperm population with JC-1 high and JC-1 low fluorescence (orange and green fluorescence, respectively, representing high and low mitochondrial activity) were determined. Samples were assessed at 24, 48, 72 and 96 h after collection.

#### 2.3.5. Sperm Chromatin Structure Assay (SCSA)

Equal volumes (50 μL) of sperm samples and buffer containing 0.01 M Tris-HCl, 0.15 M sodium chloride and 1 mM EDTA (pH 7.4; TNE) were mixed and snap-frozen in liquid nitrogen before being transferred to a −80 °C freezer for storage. Samples were taken for SCSA at 24, 48, 72 and 96 h after collection.

For evaluation, samples were thawed on crushed ice immediately before staining; 80 μL of TNE were added to 20 μL semen, 200 μL of a low-pH detergent solution containing 0.17% Triton X-100, 0.15 M NaCl and 0.08 M HCl (pH 1.2), followed 30 s later by 600 μL acridine orange (AO) (6 μg mL^−1^ in 0.1 M citric acid, 0.2 M Na_2_HPO_4_, 1 mM EDTA, 0.15 M NaCl, pH 6.0) [16]. Spermatozoa with single stranded DNA fluoresce red, whereas those with normal double stranded DNA fluoresce green. The ratio of red to (green + red) provides a measure of the proportion of spermatozoa with damaged DNA in the population (%DFI). The green and red fluorescence, as well as forward and side scatter, were measured using a FACSVerse™ flow cytometer. After collection of data, the ratio for each of the cells was calculated using FCSExpress version 2 (DeNovo Software, Thornhill, ON, Canada), and a histogram of the distribution was used to calculate %DFI.

#### 2.3.6. Bacteriology

Aliquots of the diluted samples (1 mL) on ice were sent for bacteriological culture and analysis 1 to 5 h after collection. An aliquot (1 mL) of each sample was added to 9 mL 2SP medium (0.2 mol/L sucrose in 0.02 mol/L phosphate buffer, supplemented with 10% fetal calf serum), vortexed, and serially diluted up to 1 × 10^−8^. Appropriate dilutions (0.1 mL) were plated in triplicate on Columbia Agar with 5% sheep blood, Schaedler Agar with vitamin K1 and 5% sheep blood (both BBL™, BD Diagnostics, Schwechat, Austria), and PPLO (Pleuropneumonia-Like-Organism) Agar (Difco™, BD Diagnostics, Schwechat, Austria) supplemented with 20% horse serum (Gibco™, Thermo Fisher Scientific, Vienna, Austria). Columbia Agar plates were incubated in ambient air at 33 °C, PPLO Agar at 37 °C under microaerobic conditions and Schaedler Agar at 37 °C in an anaerobic jar with gas packs (BD Diagnostics, Schwechat, Austria). Culture plates were examined daily for growth up to 96 h of incubation. Bacterial colonies were counted and mean total colony counts per sample were calculated from triplicates. The counts in each treatment were normalized to CA (i.e., expressed in relation to CA) since CA (control with antibiotics) was considered to be the industry standard.

#### 2.3.7. Statistics

Data were analyzed using PROC MIXED (repeated measures data) with stallions and ejaculates as random factors, and treatments and time as variables in the SAS software (ver. 9.4, SAS Inst. Inc., Cary, NC, USA) [17]. To test normality, diagnostic plots were used. The results are presented as Least Squares Means ± Standard Error; the differences were considered significant at level P < 0.05. Pearson correlations were made between the various parameters of sperm quality and bacterial count.

## 3. Results

### 3.1. Sperm Motility

**Figure 2:**
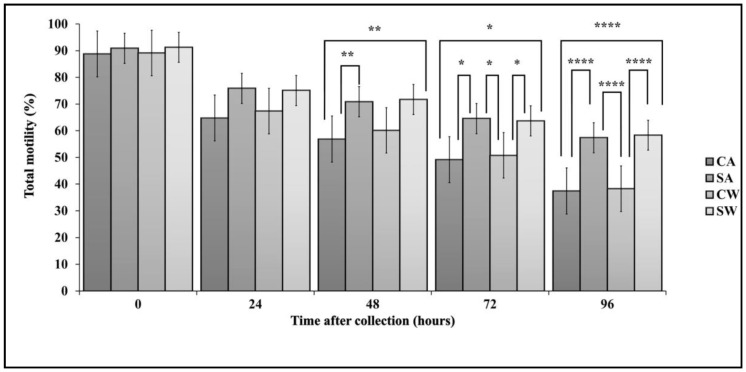
Total motility in control and MSLC samples, with and without antibiotics, during storage for 96 h at 6 °C. Values are Least Squares Means ± SE (*n* = 18). Note: * P < 0.05, ** P < 0.01, **** P < 0.0001. Abbreviations: CA, control with antibiotics; SA, modified single layer centrifugation with antibiotics; CW, control without antibiotics; SW, modified single layer centrifugation without antibiotics.

There were significant differences between treatments in TM e.g., between control and MSLC from 48 h onwards, and also with and without antibiotics (Figure 2). At each time point, TM was higher for MSLC than for control and higher for the treatments without antibiotics than with antibiotics. There was a significant decrease in TM with time for all samples.

There were significant differences between treatments for some other kinematics (Table 1): between CA and SA for, VAP and VSL at 0 h, for VCL and ALH at 24 h, for STR and LIN at 96 h; between CA and SW for VSL at 24 h, for VAP at 72 h; for VCL and ALH at 48 h; between CW and SW for VSL at 0 h and at 24 h; for VAP and VSL at 48 h, for VCL at 72 h, for STR at 96 h (P < 0.05); and between CA and SA for VCL at 0 h and for ALH at 72 h; between CA and SW for VAP, VCL, VSL, ALH at 48 h and for ALH at 72 h; between CW and SA for VAP, VCL, VSL at 0 h and for STR at 96 h; between CW and SW for VCL and ALH at 48 h (P < 0.01). Lower values for SLC were observed for VAP, VCL, VSL, ALH and STR than controls.

### 3.2. Membrane Integrity

**Figure 3:**
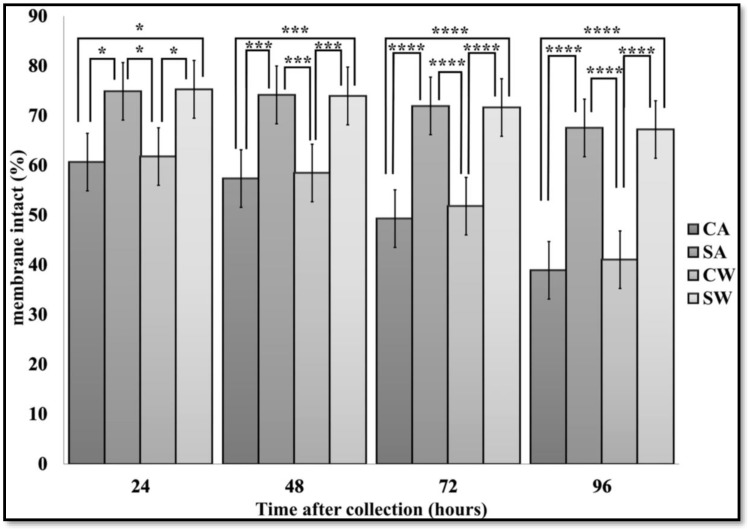
Membrane integrity in control and MSLC samples, with and without antibiotics, during storage for 96 h at 6 °C. Values are Least Squares Means ± SE (*n* = 18). Note: * P < 0.05, *** P < 0.001, **** P < 0.0001; not possible to analyze samples at 0 h. Abbreviations: CA, control with antibiotics; SA, modified single layer centrifugation with antibiotics; CW, control without antibiotics; SW, modified single layer centrifugation without antibiotics.

There were significant differences (P < 0.05) in membrane intact spermatozoa between CA and SA, CA and SW, CW and SA, CW and SW at 24 h. There were also significant differences (P < 0.001–P < 0.0001) between treatments at 48, 72 and 96 h (Figure 3). There were no differences in membrane integrity between treatments with or without antibiotics at any time points.

### 3.3. Mitochondrial Membrane Potential

The results for MMP are shown in (Table 2). Significant differences (P < 0.05) were found between CA and SA at 24 h in both MMP low and high; between CA and SW at 24 and 72 h for MMP low (P < 0.01), and at 24 and 72 h for MMP high. The SLC treatments had higher high MMP levels and lower low MMP levels than controls at each time point. There were no differences between extender with or without antibiotics at any time points.

### 3.4. SCSA

**Figure 4:**
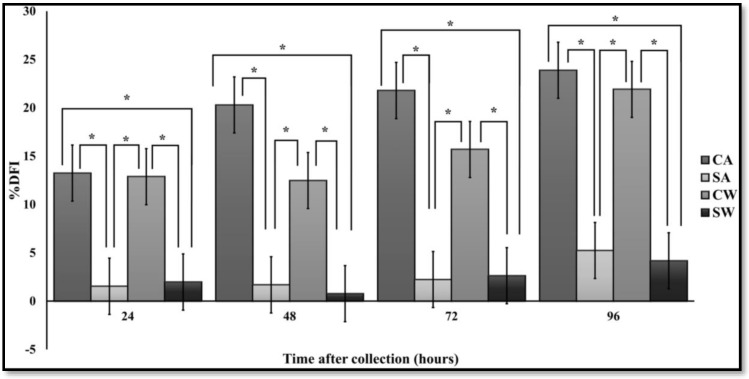
DNA fragmentation index for control and MSLC samples, with and without antibiotics (Least squares means ± SE; *n* = 18). Note: * P < 0.05; it was not possible to analyze samples at 0 h. Abbreviations: CA, control with antibiotics; SA, modified single layer centrifugation with antibiotics; CW, control without antibiotics; SW, modified single layer centrifugation without antibiotics.

Controls had significantly (P < 0.05) higher levels of %DFI than MSLC at all time points (Figure 4). There was also a significant increase in %DFI with time for control groups, whereas there was no change in chromatin damage in the MSLC groups. There were no differences between the groups with and without antibiotics.

### 3.5. Bacteriology

**Figure 5:**
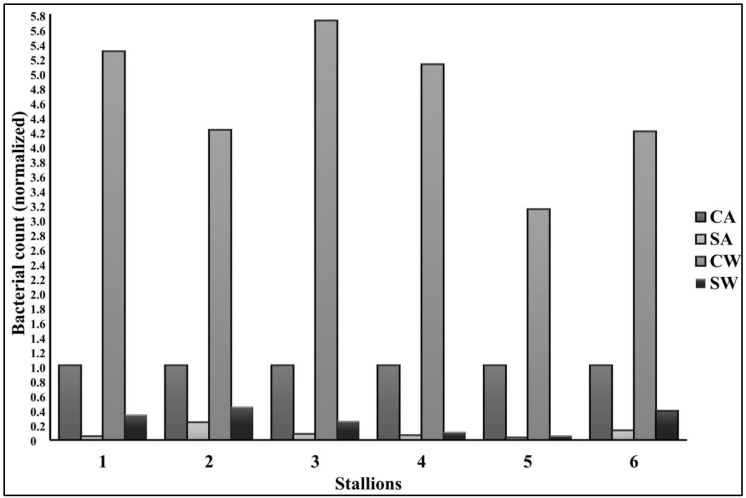
Total bacterial colony counts per treatment group relative to control with antibiotics (at 0 h); control with antibiotics has been normalized to 1 arbitrary unit. Abbreviations: CA, control with antibiotics; SA, modified single layer centrifugation with antibiotics; CW, control without antibiotics; SW, modified single layer centrifugation without antibiotics.

Figure 5 shows the proportion of total bacterial colony counts in all samples relative to CA (=1 arbitrary unit). There was a higher number of culturable bacteria in CW than in CA, but a lower number in SA and SW. The number of colonies depended on the type of plate and corresponding culture conditions (aerobic, microaerobic or anaerobic; 33 °C or 37 °C).

**Figure 6:**
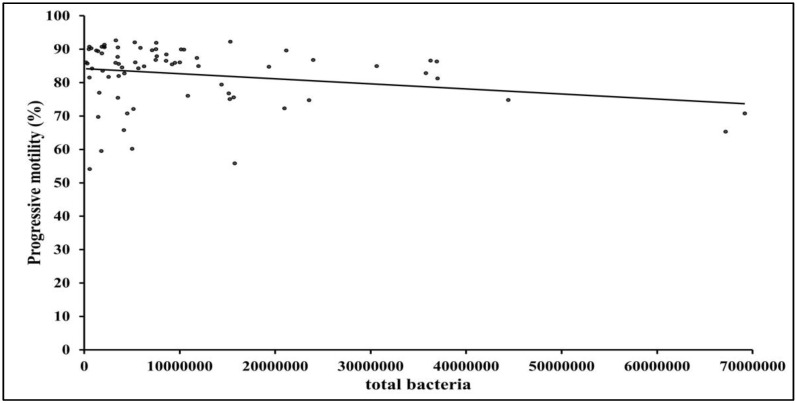
Correlation between total bacterial colony counts per sample and progressive motility at 0 h (r = −0.24; P < 0.05).

There was a significant negative correlation (P < 0.05) between total bacterial colony counts in the sample and PM (Figure 6). There were no other significant correlations between total bacterial load and sperm quality.

## 4. Discussion

The study was designed to test the effect of the presence or absence of antibiotics in the semen extender on sperm quality, and also the effect of removal of bacteria by MSLC. The presence or absence of antibiotics did not affect sperm characteristics in this study. However, MSLC did have an effect on sperm characteristics, since most parameters evaluated were higher in MSLC than in controls: e.g., TM and PM were higher in MSLC samples than in control samples, even after storage. Sperm viability was higher in MSLC samples than controls, and remained so during storage. Similarly, MMP was higher in MSLC than controls.

Our present results are in agreement with those of [18], who showed that 11 out of 30 samples of frozen commercial bull semen still contained viable bacteria despite the presence of antibiotics. Another study on stallion semen in a commercial extender demonstrated that a large number of bacteria were detectable in extended stallion semen 24–48 h after preparation [19], indicating that the presence of antibiotics does not guarantee absence of viable bacteria. In contrast, in a study on ram semen, all bacteria in the semen were sensitive to gentamicin and to ceftiofur [20]. These authors also showed that there was a significant reduction in sperm motility, velocity and viability during storage in semen containing *Escherichia coli.* Similar results were observed by [6], who noted that stallion sperm quality deteriorated during cooled storage if certain bacteria were present; this reduction in sperm quality was not altered by the presence of gentamicin.

Other studies have reported that the presence of antibiotics affects both bacteria and spermatozoa [21]. A high dose of amikacin in semen extender resulted in a decrease in PM in stallion semen [22]. A study on cooled stallion semen also showed a decrease in PM when gentamicin sulfate was included [5]. No improvement in motility parameters was reported when gentamicin was added to stored stallion semen spiked with different bacteria [6]. In buffalo (*Bubalus bubalis*) semen *Klebsiella pneumoniae*, *Staphylococcus aureus* and *Pseudomonas aeruginosa* were isolated, which were resistant to benzylpenicillin and streptomycin [23].

Several studies with colloid centrifugation have shown a reduction in bacterial contamination. Density gradient centrifugation improved sperm viability and reduced the bacterial contamination in human semen [24]. A substantial reduction in bacterial contamination was reported in boar semen samples processed by SLC [12]. In a similar study, approximately 90% of the bacterial load in stallion semen could be removed with SLC [11]. However, only 50% of the bacterial load could be removed with a higher g force [25].

The current results are in agreement with many other studies in which SLC-selected sperm samples show improved sperm characteristics in comparison to controls [26,27]. Improvements in motility and %DFI in SLC samples in stallion sperm samples compared to controls at all time points were reported by [14,27,28]. In a study with donkey semen, the use of SLC improved TM, PM, viability and normal sperm morphology after 24 h of cooled storage [29].

There was an improvement in sperm motility, sperm viability and sperm chromatin integrity in samples prepared by SLC at 0 h, 24 h and 48 h [28]. In addition, there was improved survival of stallion spermatozoa for 96 h after SLC when stored at 4 °C [30]. The present study showed similar improvements in sperm quality. Fertility was also maintained even after cooled storage for up to 96 h [31]. It is interesting to note that MSLC selected samples showed lower velocities than the controls at all time points, despite having a higher mitochondrial membrane potential, indicating higher mitochondrial activity. Similar results have been seen in other species, although the velocity measurement recorded does depend on the CASA instrument and settings used. With the SpermVision instrument, SLC-selected spermatozoa show a straighter but slower pattern of motility than controls, with less pronounced head movements than unselected spermatozoa. This motility pattern does not appear to be related to mitochondrial membrane potential, which is higher in selected spermatozoa than in controls. However, since the fertility of selected spermatozoa is higher than unselected spermatozoa [32], the lower velocity of the selected spermatozoa does not seem to reflect a problem with their ability to traverse the female reproductive tract.

According to the results of this present experiment, there was no difference in sperm quality between samples with or without antibiotics, but samples prepared with MSLC had better quality, and also lower content of bacteria than controls, even those with added antibiotics. These results indicate that MSLC can be used to improve the quality of stallion semen, and that this quality is maintained during storage regardless of whether antibiotics are included in the extender or not. These results are potentially very interesting with regard to decreasing usage of antibiotics in semen extenders although further studies are needed to determine the circumstances under which antibiotics could be excluded. Although sperm quality is thought to be improved by some other methods of sperm selection, none of these other methods has been shown to reduce bacterial contamination [32].

## 5. Conclusions

Use of MSLC with pony stallion semen improved sperm quality compared to controls, and this improvement was maintained during prolonged storage at 6 °C. The presence of antibiotics neither improved nor adversely affected sperm quality. Therefore, MSLC may be useful for controlling bacterial contamination in stallion semen but in vivo studies are needed to assess the effects on the mare.

## Figures and Tables

**Table 1:** Sperm kinematics for control and treatment groups, with and without antibiotics at 0 to 96 h (Least Squares Means ± Standard Error; *n* = 18).

Time		CA	SA	CW	SW
0 h	PM%	80.83 ± 4.62	83.60 ± 4.62	81.27 ± 4.62	84.29 ± 4.62
	VAP (µm/s)	93.97 ± 3.20 ^a^	79.02 ± 3.20 ^a,c^	95.30 ± 3.20 ^c^	82.13 ± 3.20
	VCL (µm/s)	166.14 ± 5.44 ^c^	137.84 ± 5.44 ^c,d^	163.80 ± 5.44 ^d^	143.15 ± 5.44
	VSL (µm/s)	83.75 ± 2.86 ^a^	70.76 ± 2.86 ^a,c^	85.46 ± 2.86 ^b,c^	73.29 ± 2.86 ^b^
	STR%	0.89 ± 0.01	0.89 ± 0.01	0.89 ± 0.01	0.89 ± 0.01
	LIN%	0.50 ± 0.01	0.50 ± 0.01	0.51 ± 0.01	0.50 ± 0.01
	WOB%	0.56 ± 0.01	0.56 ± 0.01	0.57 ± 0.01	0.57 ± 0.01
	ALH (µm)	3.68 ± 0.14	3.18 ± 0.14	3.74 ± 0.14	3.19 ± 0.14
	BCF(Hz)	33.90 ± 0.63	33.14 ± 0.63	34.40 ± 0.63	32.68 ± 0.63
24 h	PM%	49.74 ± 4.62	59.41 ± 4.62	52.61 ± 4.62	57.97 ± 4.62
	VAP (µm/s)	78.60 ± 3.20	68.40 ± 3.20	78.71 ± 3.20	65.59 ± 3.20
	VCL (µm/s)	144.83 ± 5.44	129.60 ± 5.44	145.28 ± 5.44	124.82 ± 5.44
	VSL (µm/s)	61.38 ± 2.86 ^a^	52.31 ± 2.86	61.91 ± 2.86 ^b^	49.32 ± 2.86 ^a,b^
	STR%	0.78 ± 0.01	0.76 ± 0.01	0.78 ± 0.01	0.75 ± 0.01
	LIN%	0.42 ± 0.01	0.40 ± 0.01	0.42 ± 0.01	0.39 ± 0.01
	WOB%	0.54 ± 0.01	0.52 ± 0.01	0.54 ± 0.01	0.52 ± 0.01
	ALH (µm)	3.91 ± 0.14	3.62 ± 0.14	3.84 ± 0.14	3.45 ± 0.14
	BCF (Hz)	31.30 ± 0.63	29.79 ± 0.63	31.27 ± 0.63	29.41 ± 0.63
48 h	PM%	44.20 ± 4.62	52.28 ± 4.62	44.92 ± 4.62	50.94 ± 4.64
	VAP (µm/s)	73.58 ± 3.20 ^a,c^	59.66 ± 3.20 ^a^	72.58 ± 3.20 ^b^	57.39 ± 3.26 ^c,b^
	VCL (µm/s)	139.63 ± 5.44 ^a,c^	114.81 ± 5.44 ^a,b^	138.72 ± 5.44 ^d,b^	111.84 ± 5.43 ^c,d^
	VSL (µm/s)	56.01 ± 2.86 ^c^	44.19 ± 2.86	55.07 ± 2.86 ^a^	41.71 ± 2.91 ^c,a^
	STR%	0.75 ± 0.01	0.73 ± 0.01	0.75 ± 0.01	0.72 ± 0.01
	LIN%	0.39 ± 0.01	0.38 ± 0.01	0.39 ± 0.01	0.37 ± 0.01
	WOB%	0.52 ± 0.01	0.52 ± 0.01	0.52 ± 0.01	0.51 ± 0.01
	ALH (µm)	3.79 ± 0.14 ^a,c^	3.20 ± 0.14 ^a,b^	3.82 ± 0.14 ^b,d^	3.10 ± 0.14 ^c,d^
	BCF (Hz)	29.11 ± 0.63	29.27 ± 0.63	29.53 ± 0.63	28.61 ± 0.63
72 h	PM%	35.13 ± 4.62	42.97 ± 4.62	37.44 ± 4.62	43.46 ± 4.62
	VAP (µm/s)	67.02 ± 3.20 ^a^	55.49 ± 3.20	65.79 ± 3.20	53.26 ± 3.20 ^a^
	VCL (µm/s)	129.66 ± 5.44	107.80 ± 5.44	128.44 ± 5.44 ^a^	104.56 ± 5.44 ^a^
	VSL (µm/s)	49.33 ± 2.86	39.17 ± 2.86	48.40 ± 2.86	37.87 ± 2.86
	STR%	0.73 ± 0.01	0.70 ± 0.01	0.73 ± 0.01	0.71 ± 0.01
	LIN%	0.38 ± 0.01	0.36 ± 0.01	0.37 ± 0.01	0.36 ± 0.01
	WOB%	0.52 ± 0.01	0.51 ± 0.01	0.51 ± 0.01	0.51 ± 0.01
	ALH (µm)	3.70 ± 0.14 ^c,d^	3.04 ± 0.14 ^c^	3.56 ± 0.14	3.00 ± 0.14 ^d^
	BCF (Hz)	27.54 ± 0.63	28.21 ± 0.63	27.86 ± 0.63	28.30 ± 0.63
96 h	PM%	25.77 ± 4.62	34.97 ± 4.62	28.52 ± 4.62	35.11 ± 4.62
	VAP (µm/s)	58.22 ± 3.20	53.72 ± 3.20	60.50 ± 3.20	51.10 ± 3.20
	VCL (µm/s)	110.36 ± 5.44	104.95 ± 5.44	121.38 ± 5.44	98.54 ± 5.44
	VSL (µm/s)	42.47 ± 2.86	37.33 ± 2.86	44.71 ± 2.86	35.84 ± 2.86
	STR%	0.73 ± 0.01 ^a^	0.69 ± 0.01 ^a,c^	0.73 ± 0.01 ^b,c^	0.69 ± 0.01 ^b^
	LIN%	0.39 ± 0.01 ^a^	0.35 ± 0.01 ^a^	0.36 ± 0.01	0.36 ± 0.01
	WOB%	0.54 ± 0.01	0.51 ± 0.01	0.49 ± 0.01	0.52 ± 0.01
	ALH (µm)	3.56 ± 0.14	3.12 ± 0.14	3.42 ± 0.14	3.00 ± 0.14
	BCF (Hz)	25.55 ± 0.63	27.89 ± 0.63	27.38 ± 0.63	27.41 ± 0.63

Note: Similar letters within rows indicate statistical difference between columns for the same parameter, ^a,b^ P < 0.05, ^c,d^ P < 0.01. Abbreviations: CA, control with antibiotics; SA, modified single layer centrifugation with antibiotics; CW, control without antibiotics; SW, modified single layer centrifugation without antibiotics; PM, progressive motility; VAP, velocity of the average path; VCL, curvilinear velocity; VSL, straight line velocity; STR, straightness, LIN, linearity; WOB, wobble; ALH, lateral head displacement; BCF, beat cross frequency.

**Table 2:** Mitochondrial membrane potential for control and treatment groups, with and without antibiotics (Least Squares Means ± Standard Error; *n* = 18).

**JC-1 Low %**	**CA**	**SA**	**CW**	**SW**
24 h	48.97 ± 6.63 ^a,b^	34.60 ± 6.63 ^a^	46.83 ± 6.63	33.61 ± 6.66 ^b^
48 h	55.96 ± 6.63	51.70 ± 6.63	56.03 ± 6.63	48.74 ± 6.63
72 h	69.64 ± 6.63 ^a^	59.11 ± 6.63	64.93 ± 6.63	55.37 ± 6.63 ^a^
96 h	77.23 ± 6.63	66.14 ± 6.63	73.11 ± 6.63	66.88 ± 6.63
**JC-1 high %**				
24 h	48.92 ± 6.63 ^a,b^	62.96 ± 6.63 ^a^	50.87 ± 6.63	64.05 ± 6.63 ^b^
48 h	41.85 ± 6.63	46.65 ± 6.63	42.22 ± 6.63	49.36 ± 6.66
72 h	29.15 ± 6.63 ^b^	39.83 ± 6.63	34.14 ± 6.63	43.59 ± 6.63 ^b^
96 h	21.83 ± 6.63	33.16 ± 6.63	26.27 ± 6.63	32.40 ± 6.63

Note: Similar letters indicate statistical difference between columns at the same time point, ^a^ P < 0.05, ^b^ P < 0.01. Abbreviations: JC-1 low, low Mitochondrial Membrane Potential; JC-1 high, high Mitochondrial Membrane Potential. It was not possible to analyze samples at 0 h.
